# Cytotoxic and Antitumor Activity of Sulforaphane: The Role of Reactive Oxygen Species

**DOI:** 10.1155/2015/402386

**Published:** 2015-06-22

**Authors:** Piero Sestili, Carmela Fimognari

**Affiliations:** ^1^Department of Biomolecular Sciences, University of Urbino “Carlo Bo”, 61029 Urbino, Italy; ^2^Department for Life Quality Studies, Alma Mater Studiorum-University of Bologna, 47921 Rimini, Italy

## Abstract

According to recent estimates, cancer continues to remain the second leading cause of death and is becoming the leading one in old age. Failure and high systemic toxicity of conventional cancer therapies have accelerated the identification and development of innovative preventive as well as therapeutic strategies to contrast cancer-associated morbidity and mortality. In recent years, increasing body of *in vitro* and *in vivo* studies has underscored the cancer preventive and therapeutic efficacy of the isothiocyanate sulforaphane. In this review article, we highlight that sulforaphane cytotoxicity derives from complex, concurring, and multiple mechanisms, among which the generation of reactive oxygen species has been identified as playing a central role in promoting apoptosis and autophagy of target cells. We also discuss the site and the mechanism of reactive oxygen species' formation by sulforaphane, the toxicological relevance of sulforaphane-formed reactive oxygen species, and the death pathways triggered by sulforaphane-derived reactive oxygen species.

## 1. Introduction

According to recent estimates, cancer continues to remain the second leading cause of death and is becoming the leading one in old age [1]. It is projected that by 2030 the number of new cancer cases will increase by 70% worldwide due to demographic changes alone [2]. Lack of effective diagnostic tools for early detection of several tumors, limited treatment options available to patients with advanced stages of cancer, and onset of multiple drug resistance favor poor prognosis and high mortality rate. The moderate improvement of survival, severe toxicity profile, and high costs that characterize many current anticancer therapies indicate that a threshold in terms of clinical benefit and tolerance in patients has been reached and advocate the identification and development of innovative preventive as well as therapeutic strategies to contrast cancer-associated morbidity and mortality.

Epidemiological, preclinical, and clinical studies have generally concluded that a diet rich in phytochemicals can reduce the risk of cancer [2]. Due to their antioxidant, anti-inflammatory, and antiproliferative activities as well as modulatory effects on subcellular signaling pathways, fruits and vegetables, which contain a diverse range of phytochemicals, are suggested to protect against cancer incidence and mortality [3–5]. Plants constitute a primary and large source of various chemical compounds including alkaloids, flavonoids, phenolics, tocopherols, organic acids, triterpenes, and isothiocyanates. Belonging to the Cruciferae family, broccoli, cauliflower, cabbage, kale, Brussels sprouts, and radish have been linked to the high content of secondary metabolites and multipharmacological functions [6]. Clinical and preclinical studies have actually reported that cruciferous vegetables exert anticarcinogenic, anti-inflammatory, and antioxidant activities largely attributed to their content of many bioactive components including flavonoids such as quercetin, minerals such as selenium, and vitamins such as vitamin C [7–9]. However, glucosinolates are the most studied bioactive compounds in crucifers associated with cancer protection. They are characterized by a basic structure containing a *β*-D-thioglucose group, a sulfonated oxime group, and a side chain derived from methionine, phenylalanine, tryptophan, or branched-chain amino acids. Of note, glucosinolates are not bioactive until they have been transformed to a chemically related isothiocyanate (ITC) by a hydrolytic reaction catalyzed by the endogenous enzyme myrosinase. The hydrolytic reaction takes place when myrosinase is released by disruption of the plant cell during harvesting, processing, or chewing of cruciferous vegetables or if the plant myrosinase has been denatured by cooking and by bacterial myrosinase in the human colon. One of the most promising and characterized anticancer ITCs is sulforaphane (SFR), generally found as glucoraphanin in high concentrations in broccoli.

SFR is passively absorbed by cells, where it is rapidly conjugated with glutathione (GSH) by glutathione S-transferases (GSTs). Then, it is metabolized sequentially by *γ*-glutamyl-transpeptidase, cysteinyl-glycinease, and N-acetyltransferase, and the derived conjugates are transported into the systemic circulation. The major urinary excretion products are mercapturic acid and cysteine conjugate forms [10].

In blood, SFR can achieve *μ*molar concentrations and accumulate in tissues [11]. Rat treatment with a single oral dose of 50 *μ*mol of SFR leads to a peak plasma concentration of about 20 *μ*M [12]. However, after dietary consumption, SFR levels in humans are lower and closer to 3 *μ*M [13].

SFR administered orally protects against animal carcinogenesis and induces antiproliferative effects in human tumor cells in xenograft models. Mechanisms of cancer chemoprevention by SFR are diversified and include the alterations of carcinogen metabolism through the induction of Nrf2-regulated genes of Phase-II detoxification enzymes (glutathione S-transferase, quinone reductase, glucuronosyltransferase, etc.) and the inhibition of Phase-I enzymes that activate toxic chemical compounds, thus lowering the levels of the carcinogens interacting with DNA [14]. Of note, the inducer activity was also reported in humans. A placebo-controlled dose escalation study demonstrated that dietary SFR-containing broccoli sprout extracts upregulate mRNA levels for Nrf2-dependent enzymes (heme oxygenase 1, NAD(P)H:quinone oxidoreductase-1, and glutathione transferases) in nasal lavage [15].

A second important antitumor mechanism is the ability of SFR to block cell proliferation and induce apoptosis of cancer cells, thus reducing tumor growth. A plethora of different and partly dependent molecular mechanisms mediates its cytostatic and cytotoxic activities including mitogen-activated protein kinase (MAPK) responses, nuclear factor-*κ*B (NF-*κ*B) activity, conformational and functional changes of tubulin, microtubule disruption, tubulin precipitation, and degradation of both *α*-tubulins and *β*-tubulins, enhancement of proteasomal activity, and modulation of Bax : Bcl-2 ratio [5, 16–21]. As an example, an increase in intracellular free Ca^2+^ was detected in glioblastoma cells following treatment with SFR, suggesting the activation of Ca^2+^-dependent pathways for apoptosis, such as upregulation of calpain, a Ca^2+^-dependent cysteine protease, and increased Bax : Bcl-2 ratio [22]. Other studies reported that SFN-induced apoptosis was associated with p53 gene activation [23].

The modulation of epigenetic marks is a third mechanism that has been suggested to be involved in the anticancer activity of SFR. SFR is actually able to enhance global histone acetylation through the inhibition of histone deacetylase activity [24]. Accordingly, an increase at the* bax* and the* p21* promoter regions has been detected in animal models [25]. It is worth mentioning that this effect has been observed also in healthy human subjects, where a single dose of 68 g of broccoli sprouts reduced histone deacetylase activity in peripheral blood mononuclear cells 3 and 6 h after consumption [25].

Herein, we highlight that SFR cytotoxicity derives from complex, concurring, and multiple mechanisms, among which the generation of reactive oxygen species has been identified as playing a central role in promoting apoptosis and autophagy of target cells. Furthermore, we critically review the scientific knowledge about the site and the mechanism of reactive oxygen species (ROS) formation by SFR, the toxicological relevance of SFR-formed ROS, and the death pathways triggered by SFR-derived ROS.

## 2. ROS Signaling in Cancer

Oxidative stress plays a role in many clinical conditions such as cancer, diabetes, atherosclerosis, chronic inflammation, viral infection, and ischemia-reperfusion injury [26]. In particular, cancer is generally associated with a prooxidative shift in the redox state. Since cancer patients often present reduced glucose clearance capacity, high glycolytic activity, and lactate production, it has been suggested that the observed prooxidative shift is mediated by an enhanced availability of mitochondrial energy substrate [27]. ROS can favor mutagenesis, tumor promotion, and progression. Indeed, they are able to induce DNA and protein damage, damage to tumor suppressor genes, and increased expression of protooncogenes [28]. Damage to DNA by ROS has been widely accepted as a major cause of cancer [29]. In patients with pathologies associated with a risk of cancer such as Fanconi's anemia, chronic hepatitis, or cystic fibrosis, an increased rate of oxidative DNA damage or deficient DNA repair system has been observed [30–33]. The ROS-induced mutations include a range of specifically oxidized purines and pyrimidines, alkali labile sites, single-strand breaks, and instability formed directly or by repair processes [34]. Although all the four DNA bases can be modified by ROS, mutations mainly involve modification of GC base pairs, while AT base pair mutations are rarely observed [35]. In humans, G → T transversions are the most frequent mutations observed in the p53 suppressor gene of tumor cells [36]. High levels of mutated bases observed in neoplastic tissues may be due to the production of large amount of H_2_O_2_ [37].

Accordingly, oxidative DNA damage has been suggested to be involved in the development of many different cancers. Increased steady-state levels of 8-oxo-dG adducts have been observed in inflamed breast cancer tissues, where malignant progression can occur [38], and in hepatocellular carcinoma [39]. Hepatocarcinoma is often associated with hepatitis B or hepatitis C virus infection or ingestion of food contaminated by aflatoxins [40, 41]. Hepatitis B or hepatitis C virus-induced oxidative stress is causally associated with the genesis of hepatocarcinoma [31, 42] and G → T transversion is a common type of mutation caused by aflatoxins [43]. Also the high incidence of prostatic carcinoma in men aged > 50 years, the paucity of chemicals causally linked to the onset and development of this specific tumor, and the increased ROS production by mitochondria detected in aged tissues [44] led to hypothesizing an association between prostate cancer and endogenously formed genotoxins that accumulate in later life like ROS [45].

The proproliferative effects of ROS are related to redox-responsive cell signaling cascades, and sometimes increased proliferation and expression of growth-related genes are observed even in normal cells if exposed to H_2_O_2_ or O_2_^−∙^. Although the role of ROS in cell growth regulation is cell-type specific and dependent upon the form of the oxidant as well as the concentration of the particular ROS, the modification of gene expression by ROS has been found to affect cell proliferation and apoptosis through the activation of transcription factors including MAPK, AP-1, and NF-*κ*B pathways. Likewise, ROS can function as second messengers and activate NF-*κ*B by tumor necrosis factor and cytokines [26].

Finally, oxidative stress is involved in malignant transformation. Epithelial-mesenchymal transition, characterized by loss of cell-cell junctions, polarity and epithelial markers, and acquisition of mesenchymal features and motility, has been suggested to be involved in cancer progression and metastasis [46]. Recently, it has been found that matrix metalloproteinases cause epithelial-mesenchymal transition associated with malignant transformation* via* a pathway dependent upon production of ROS [47].

## 3. Putative Role of ROS in the Cytotoxic and Anticancer Activity of SFR

The term hormesis is used to describe the apparently paradoxical phenomenon in which a specific compound induces biologically opposite effects depending on its concentration: in particular, there is a stimulatory or beneficial effect at low doses and an inhibitory or toxic effect at higher ones. Today there is general consensus on the fact that SFR (and some other ITCs) can be considered as a hormetic moiety; that is, at low doses it exerts chemopreventive, indirect antioxidant, and cytoprotective effects, while at higher doses it exhibits cytotoxic and antitumor properties [48, 49]. This scenario paves the way to a double exploitation of SFR in cancer, as a chemopreventive agent to reduce the onset of tumors through diets enriched in functional foods, as well as a direct antineoplastic agent at higher dosage regimens more reliably attainable through pharmaceutical delivery of purified SFR [16].

Most of the studies have been aimed at elucidating the chemopreventive activity of SFR, which, as above reported, has been attributed to its indirect antioxidant capacity involving the activation of Phase-II detoxification enzymes and the inhibition of Phase-I enzymes [16]: in this light, SFR acts strengthening the cellular defenses against oxidative damage and promoting the removal of carcinogens.

However, increasing attention has been devoted to the cytotoxic and anticancer activity since the discovery of SFR antitumor effects in pancreatic carcinoma cells and other tumor cell lines [11, 16, 50–52]. Notably, all the studies dealing with SFR toxicity report that these effects occur at concentrations above 5–10 *μ*M, that is, levels which can be barely maintained through cruciferous diet intake [23].

**Figure 1:**
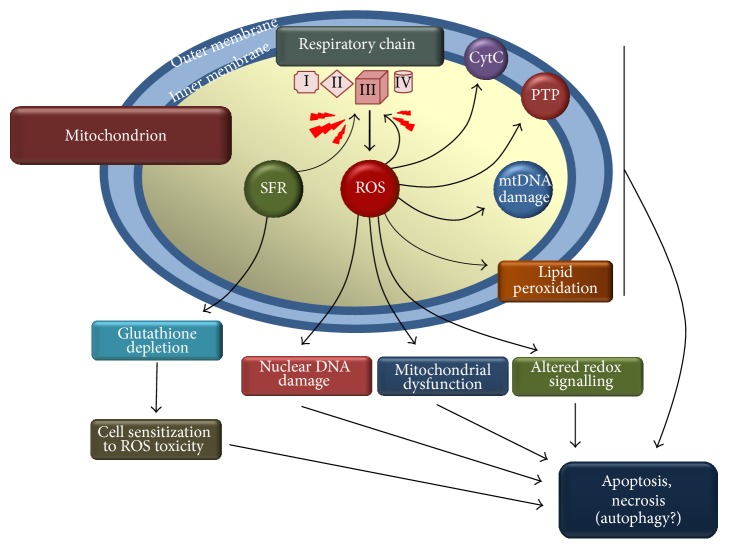
Schematic representation of the mitochondrial ROS formation elicited by sulforaphane. Sulforaphane (SFR) inhibits mitochondrial respiratory chain at the level of Complex III in an antimycin-like fashion. ROS initiate a cascade of toxic events culminating in apoptosis, necrosis, and, although still to be fully demonstrated, autophagy. Arrows indicate the toxicologically relevant events elicited by ROS. CytC: cytochrome C leakage; mtDNA: mitochondrial DNA; PTP: permeability transition pore opening.

SFR cytotoxicity seems to derive from complex, concurring, and multiple mechanisms [5, 16, 17, 22, 23, 53, 54]. Among these mechanisms, the generation of ROS is important in promoting apoptosis and autophagy of target cells [55, 56]. Indeed Singh et al. [57] reported that high concentrations of SFR caused extensive death in prostate cancer cells, an effect which could be prevented by catalase overexpression. ROS generation in SFR-treated cells was accompanied by disruption of mitochondrial membrane potential, cytosolic release of cytochrome C, cleavage of poly-ADP-ribose polymerase, and apoptosis [57] (Figure 1). ROS generation is* per se* a potentially toxic phenomenon, but it is important noting that cells treated with high doses of SFR undergo a situation of increased ROS sensitivity since a peculiar capacity of the isothiocyanate consists in depleting the GSH cellular pool [58, 59] (Figure 1), an effect which is particularly severe with high, supranutritional SFR concentrations. Indeed, depletion of GSH deprives cells of a first line, soluble antioxidant defense [53, 60, 61], giving rise to a “vicious oxidative cycle” (ROS production in cells which at the same time are being depleted in GSH) which is indirectly demonstrated by the fact that N-acetylcysteine (NAC) supplementation enhanced cell survival opposing to GSH depletion [57, 58] rather than acting itself as a mere, direct antioxidant.

An important issue arising from the above studies is where and how ROS are formed. As to the site and the mechanism of ROS formation by SFR, it was noted that mitochondrial respiratory complex inhibitors prevented SFR-caused ROS generation, an event which was paralleled by increased cell survival [56, 57]. Similarly, cells with respiration deficient phenotype were significantly less sensitive as compared to respiratory proficient, wild-type cells, and they did not produce ROS upon SFR treatment [56, 57]. SFR-caused ROS have been detected and visualized in cultured cells by means of specific dyes such as dihydrorhodamine or dihydrodichlorofluorescein which fluoresce upon oxidation by ROS: importantly the conditions described above (i.e., the use of respiratory complexes inhibitors, cells bearing respiratory deficient phenotype) prevented the oxidation of these dyes caused by SFR. Thus, mitochondria are likely to represent the site where SFR promotes ROS generation (Figure 1). This finding is in keeping with the observation that loss of mitochondrial transmembrane potential, release of cytochrome C, and mitochondrial damage are in effect induced by SFR and other ITCs. The molecular interaction of SFR with mitochondria has also been studied: it appeared that SFR is capable of inhibiting, probably* via* electrophilic interactions with specific SH residues, mitochondrial respiratory chain Complex I, Complex II, and Complex III [56]. However, although confirming Complex I, Complex II, and Complex III inhibition, a more recent study by our group indicated that the major and likely most crucial inhibition affects Complex III [54]. Indeed, using pharmacological inhibitors of respiratory complexes, we showed that rotenone, but also myxothiazol, prevented ROS formation in SFR intoxicated Jurkat leukemia cells. Rotenone is likely to hamper ROS formation in SFR-treated cells because, as a Complex I inhibitor [62], it impedes at the origin the electron flow to other, possibly pivotal targets located downstream, such as Complex III. Indeed, myxothiazol, which was as effective as rotenone in preventing SFR-derived ROS, is a selective Complex III inhibitor [63, 64], which blocks the electron flow through this complex and, importantly, the accumulation of ubisemiquinone (see below). Its efficacy indirectly demonstrates that SFR (at least up to 30 *μ*M, the highest concentration tested in this study) does not affect electron flow through the respiratory chain upstream to Complex III since, if electrons do not reach this site, myxothiazol would not prevent SFR-caused ROS formation. Notably, chemical inhibition of Complex III by agents acting as antimycin A (i.e., differently from myxothiazol) is known to represent a common and toxicologically relevant mechanism capable of boosting ROS generation within mitochondria [65–68]. Indeed, this latter mode of inhibiting Complex III, which is likely shared by SFR, causes an accumulation of ubisemiquinone which starts serving as an electron donor for molecular oxygen in a reaction producing superoxide anion and its dismutation product H_2_O_2_, which undergoes Fenton reaction and finally attacks sensitive cellular targets.

Thus, SFR does not undergo any direct oxidation/reduction reaction leading to ROS or radical species by-products but rather promotes the onset of mitochondrial events culminating in ROS formation through its antimycin-like Complex III inhibitory properties.

The next important issues refer to the toxicological relevance of mitochondrially formed ROS and to which death pathways are triggered by SFR-derived ROS.

One of the most sensitive cellular targets of ROS is nuclear DNA, where ROS cause extensive damage. Evidence of some genotoxic activity of SFR and other ITCs had already emerged, but the first study investigating the DNA damaging activity of SFR was that by Sekine-Suzuki et al. [69] reporting that SFR induces DNA double strand breaks in the nuclear DNA of HeLa cervical cancer cells. However, these authors did not investigate the mechanism of the DNA damaging effect of SFR and they probably detected secondary DNA fragmentation due to the ongoing apoptosis caused by the ITC rather than frank DNA lesions: indeed the exposure times to SFR were too long (24 h, i.e., a time conceivable with the onset of apoptosis) as compared to the kinetic of ROS formation in SFR intoxicated cells (1–3 h). In our previously cited study [54], we specifically addressed the relationship between ROS formation and DNA damage in SFR-treated human leukemia and umbilical vein endothelial cells. We found that SFR causes DNA single strand breaks (i.e., the type of lesion typically induced by ROS, unlike double strand breaks which are generated only in the presence of very high ROS concentrations [70]) with a kinetic (1–3 h) which paralleled that of ROS formation in SFR-treated cells. Furthermore, it was found that all the conditions blocking the mitochondrial respiratory chain and in particular myxothiazol (see above), or quenching ROS by means of the iron chelator* o*-phenanthroline, prevented DNA damage. These findings clearly indicate that ROS, produced* via* the antimycin-like interaction of SFR with mitochondrial respiratory chain at the Complex III level and then diffusing within the nucleus, are responsible for the observed DNA lesions.

Recent observations have extended our knowledge on SFR interactions with DNA homeostasis since, besides its ROS-mediated DNA damaging capacity, SFR was also shown to inhibit DNA repair processes [49, 71]. SFR sensitized HeLa cells to X-irradiation [71], and the radiosensitization was ascribed to the capacity of SFR of inhibiting the two major processes of DNA double-strand breaks repair (DNA double-strand breaks are a highly toxic DNA lesion typically and efficiently caused by ionizing radiations), namely, homologous recombination repair and nonhomologous end joining. Accordingly, other authors found that high SFR concentrations decrease the expression of a number of DNA repair genes [49] and inhibit nuclear excision repair* via* abstraction of zinc from the xeroderma pigmentosum A (XPA) protein [72]. The ROS dependence of these effects has not been addressed, but the picture arising from this further notion is indicative of a marked pleiotropism of the SFR-DNA interactions since SFR is simultaneously capable of damaging DNA [49, 54, 73, 74], inhibiting DNA repair [49, 71], and finally sensitizing cells to established anticancer agents such as X-rays [71] or doxorubicin [49] mostly acting through a DNA damaging action.

DNA damage, depending on its level and persistence, might promote cell death: indeed DNA lesions are recognized as efficient proapoptotic stimuli. Hence, ROS-dependent DNA breaks are likely to contribute to SFR-induced apoptosis which, in fact, is the type of cell death caused by SFR. Many authors have investigated the role of ROS in SFR-induced apoptosis and they invariably reported that ROS generated within mitochondria contribute to or are fully responsible for the apoptotic response [18, 75]. Besides DNA damage, the proapoptotic events which have been attributed to SFR-caused ROS are the collapse of mitochondrial membrane permeability [57, 61, 74], activation of caspase-3 and caspase-9 [57, 61, 76], downregulation of antiapoptotic Bcl-2 expression [77], Bax and p53 gene activation [23, 77], and G2/M phase cell cycle arrest [56] and have been observed in a wide variety of heterogeneous cell lines.

SFR has been shown to induce autophagy in colon and prostate cancer cells and more recently in pancreatic cells [78]: in this cell line, SFR induces autophagy* via* a ROS-dependent mechanism. SFR, at supranutritional and cytotoxic concentrations (20 or 60 *μ*M for 24 h), induced a significant increase of autophagosome formation as well as of other reliable markers of autophagy, and all these effects could be prevented by NAC cotreatment, suggesting that this response is causally related to ROS production or depletion of GSH, that is, two prooxidative events. Modulation of autophagy with specific inhibitors (rapamycin or chloroquine) did not affect, however, cell survival in SFR-treated cells, suggesting that, at least in this cell system, autophagy does not concur to the actual cytotoxic activity of SFR itself. On the contrary, in other cell systems (colon and prostate cancer cells) induction of autophagy by SFR seems to exert a cytoprotective effect [79, 80] but, unfortunately, the ROS dependence of the autophagic process had not been investigated in these studies. Thus, although the ROS dependence of SFR-induced autophagy has been demonstrated by Xiao et al. [56] and Naumann et al. [78], the problem of its sensitizing* versus* protective relevance in SFR-induced cytotoxicity is not yet clear and needs further investigations.

Taken collectively, the above reports unequivocally suggest that mitochondrial production of ROS is an important event in high SFR concentrations cytotoxicity. However, there is not clear consensus on the relative contribution of ROS-dependent mechanisms to SFR toxic capacity. Indeed, some reports show that abrogating ROS production or quenching ROS almost completely protects target cells from SFR killing [56, 57, 77], while another [54] found that similar conditions granted only a partial protection. One possible explanation may relate to cell-type specific effects: indeed different cell lines have been used in these studies. Another possible explanation is that some studies interpreted the full prevention of SFR cell killing by NAC as a neat antioxidant effect; that is, acting as an antioxidant NAC quenches ROS-derived SFR thus abrogating SFR toxicity. However, it is of worth that preloading of cells with fairly high doses (2–10 mM) of a –SH-bearing compound such as NAC is likely to prevent also the fall of GSH stores [58] in cells exposed to the comparatively low doses (1–30 *μ*M) of SFR used in these studies: in this light, the full protection of NAC would not be solely dependent on the presumed quantitative quenching of SFR-caused ROS, but rather it would reflect the cumulative effect of GSH preservation plus that of ROS scavenging. Indeed, another study showed that an established antioxidant which cannot, unlike NAC, serve as GSH repletive, namely,* o*-phenanthroline, abrogated ROS production and the ensuing DNA damage but did not completely protect cells from SFR toxicity [54]. Similar results were obtained with inhibitors of mitochondrial respiratory chain [54]. Interestingly, all these conditions prevented SFR-induced ROS but did not prevent the fall in cellular GSH, an effect which, on the contrary, is likely to be afforded by NAC. In this light, the catastrophic depletion of cellular GSH caused by high SFR concentrations would represent* per se* another cytotoxically relevant phenomenon. In addition, this same event may act additively or synergistically with ROS with its own contribution to generate oxidatively stressing/sensitizing conditions, that is, the “*vicious oxidative cycle*” described at the beginning of this chapter: notably, Han et al. [81] showed that, below certain threshold levels, mitochondrial GSH depletion increases ROS production and diffusion under conditions of Complex III inhibition.

That ROS are not the only mediators of SFR toxicity is also suggested by the finding that SFR inhibits protein synthesis in human prostate cancer cells* via* a ROS insensitive mechanism [82].

A selective toxicity of SFR to cancer cells has been demonstrated in different experimental models. As an example, SFR induces cytotoxic and cytostatic effects in different prostate cancer cell lines, but not in their normal counterpart [83]. On the basis of different observations, several studies strongly suggest a role of ROS in the selective toxicity to cancer cells of SFR. SFR-induced ROS causes membrane lipid peroxidation and generation of 4-hydroxynonenal [84] and apoptotic signals generated by SFR can be abrogated by inhibiting SFR-induced lipid peroxidation and accumulation of 4-hydroxynonenal. This evidence supports the pivotal role that 4-hydroxynonenal plays in the biological activity of SFR [84]. 4-Hydroxynonenal is an important second messenger involved in signaling for cell proliferation and apoptosis and in regulating gene expression in different cell types [85–90]. In particular, it evokes dichotomous effects through the activation of the defense mechanisms against oxidative stress, such as Nrf2 and heat shock factor 1 [91–93], at low concentrations and the induction of apoptosis at higher, supraphysiologic ones [94]. Thus, it is possible that similar dichotomous effects of SFN are responsible for its differential effects on normal and cancer cells. The ability of SFR to generate ROS and oxidative stress in cells leading to the activation of proapoptotic signaling and simultaneously activate defense mechanisms, such as Nrf2, that protect against oxidative stress and its intrinsic toxicity is consistent with the above reported hypothesis. Generation of 4-hydroxynonenal upon cell exposure to SRF could therefore represent an event implicated in its selective effects on cancer cells.

## 4. Conclusion

Although further studies are needed to clarify the relative importance of ROS in SFR toxicity, the effects which tie to ROS generation are definitely important for the ITC's cytotoxic activity and may represent the bases for its rationale exploitation in cancer therapy as a single agent or in association with other antineoplastic agents or drugs to potentiate their anticancer efficacy.
